# Damage response signaling by the extracellular adenosine pathway: control of infection outcome during host aging

**DOI:** 10.1128/msphere.00640-23

**Published:** 2025-08-29

**Authors:** Manmeet Bhalla, Shaunna R. Simmons, Alexsandra Lenhard, Anagha Betadpur, Michael C. Battaglia, Elsa N. Bou Ghanem

**Affiliations:** 1 Department of Microbiology and Immunology, University at Buffalo School of Medicine 192705, , Buffalo, New York, USA; University of Kentucky College of Medicine, Lexington, Kentucky, USA

**Keywords:** neutrophils, aging, pneumonia, lung infections, immunosenescence, damage signaling, adenosine, CD39, CD73, timing

## Abstract

In response to damage triggered by various stimuli including infections, ATP is released from damaged cells and converted to adenosine in the extracellular space by the ectonucleotidases CD39 and CD73. Extracellular adenosine is an immune modulatory molecule that signals via four G-protein receptors: A1, A2A, A2B, and A3, which can have opposing downstream effects on immune responses. In this minireview, we follow up on our mSphere of Influence commentary that focused on the A2B receptor (2019) to give a broader view of the role of the extracellular adenosine signaling pathway in host defense against infections. Studies demonstrate that extracellular adenosine serves as a key signaling molecule regulating the balance between effective pathogen clearance and immunopathology during infection. Extracellular adenosine displays dose- and time-dependent roles during infection, with individual adenosine receptors playing specific roles in controlling immune responses. Age-driven changes in this pathway contribute to the increased susceptibility of older hosts to certain infections, although there are several key unanswered questions about the role of the extracellular adenosine pathway in immunosenescence. Clinical and translational findings reveal a role for extracellular adenosine production and signaling in infections in humans, and there have been recent advances, but several ongoing challenges remain in pharmacologically targeting this pathway to reshape host immune responses.

## INTRODUCTION

### The role of damage in infection outcome

Individuals display heterogeneity in overall infection outcomes ranging from infection resistance all the way to overt disease and death (1–3). Overall infection outcomes are governed by the ability of the host to clear the infectious organism or tolerate the organism without illness and finally recover from any resulting infection sequelae (4–6). These outcomes are proposed to be governed by the damage response, a framework proposed by Liise-Anne Pirofski and Arturo Casadevall, that postulates that the result of an infection is shaped by the amount of overall damage caused by factors produced by the infectious organism and the host immune response (6). This leads to the question of what exactly is host damage, and how does it shape infection outcome? Organ pathology and function are traditional measures of damage. However, certain molecules termed damage-associated molecular patterns (DAMPs) act as signals that activate immune responses (7, 8). These are host-derived endogenous molecules that are produced or released upon cell damage that is triggered by multiple stimuli such as injury, radiation, toxic drugs, and infection (7, 8). DAMPs are heterogeneous in nature and include nucleic acids, proteins, glycans, and metabolites (9, 10). Cellular damage can result in a change in location, concentration, or alteration of these molecules, converting endogenous compounds into DAMPs that act as a danger signal, indicating disruption of homeostasis.

### The extracellular adenosine pathway

**Fig 1:**
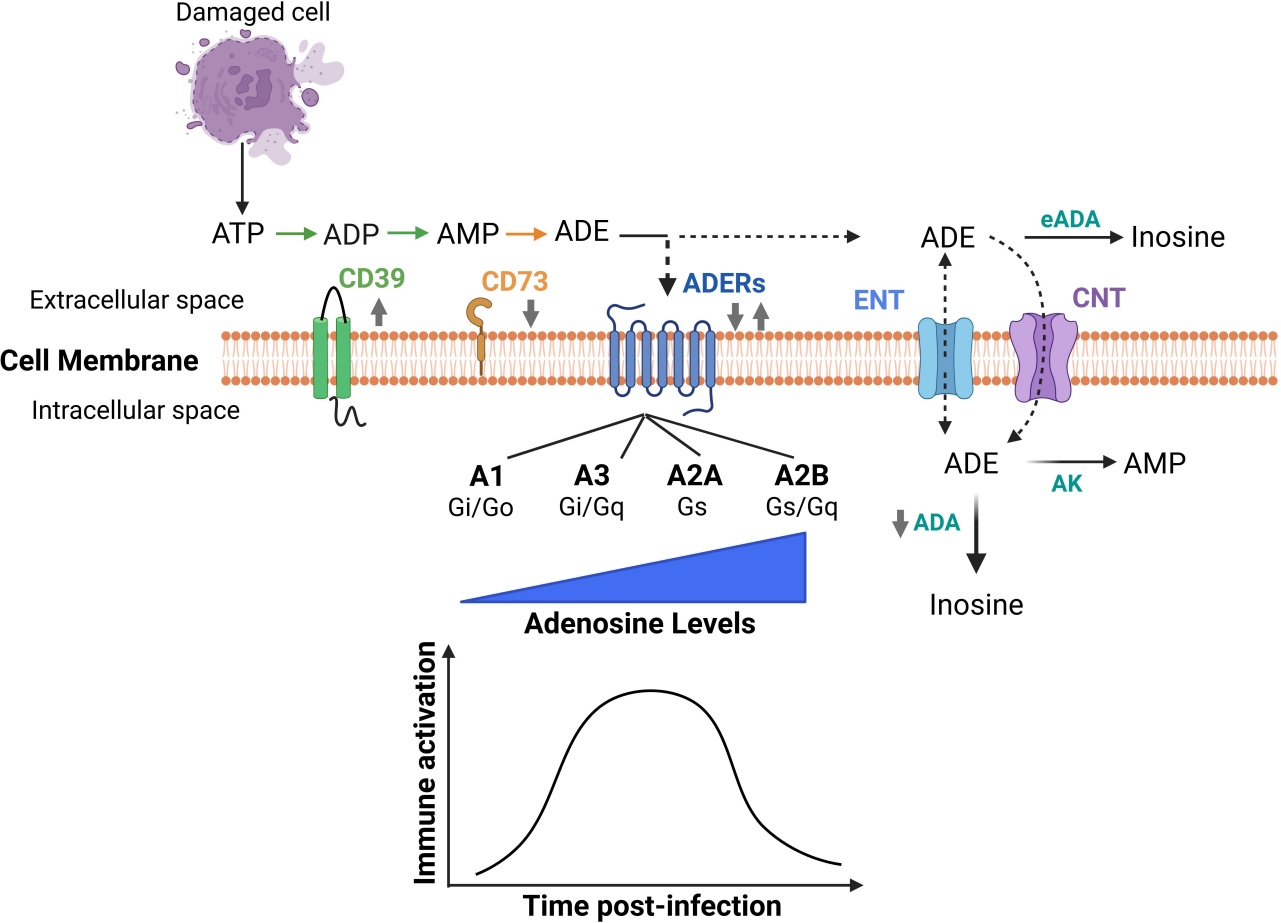
The extracellular adenosine signaling pathway. Signaling via adenosine receptors plays a time-dependent role in immune activation and host defense. The gray arrows indicate changes with aging. Human T cells display ↑ A2A mRNA, ↓ CD73, ↑ CD39, and ↓ responsiveness to ADE. Human peripheral blood lymphocytes display ↑ CD73 mRNA, ↑ CD39, and ↓ ADA. Human T cells in COVID-19 display ↑ CD39 on human regulatory T cells (Tregs), ↑ CD39^+^ CD4 T cells, and ↓ % CD73^+^ CD8 T cells. Murine polymorphonuclear cells (PMNs) display ↓ CD73 and ↓ ADE production. Murine cells/tissues in infection display ↓ A1 receptor (A1R), A2A receptor (A2AR), A2BR, and A3R on circulating and pulmonary PMNs, and ↓ CD73 on lung tissues during *Streptococcus pneumoniae* infections; ↑ CD73 on Tregs during *Listeria monocytogenes* infections; failure to upregulate A2AR on intestinal tissues during Candida infections. ADE: adenosine; eADA: extracellular ADA; AK: adenosine kinase; ADERs: adenosine receptors; ENT: equilibrative nucleoside transporter; CNT: concentrative nucleoside transporter; Gi/q/o/s: G protein types coupled to the receptors. Graphics made in BioRender.

We propose that extracellular adenosine, which is adenosine that is made and signals in the extracellular environment, is a DAMP that mediates damage response signaling within the host. Upon cellular damage, ATP is released from the intracellular compartment to the outside and converted to adenosine by the sequential action of two ectonucleotidases, CD39, which converts ATP to ADP and then AMP, and CD73, which de-phosphorylates AMP to adenosine (Fig. 1) (11). While ATP and ADP are classified as DAMPs (7, 8), adenosine is often overlooked and not included in the DAMP classification, although it fits the definition perfectly. At baseline, in the absence of disease, levels of extracellular adenosine, which is a purine nucleoside, and typically made within the cell, are very low/undetectable. Adenosine levels in the extracellular space are tightly controlled by transporters, including passive equilibrative nucleoside transporters that allow bi-directional movement of adenosine based on its own gradient, as well as concentrative nucleoside transporters that couple sodium influx with uptake of adenosine into cells (Fig. 1) (12). Adenosine can be broken down by adenosine deaminase (ADA), which is expressed within the cell or on the cell surface, and converts adenosine to inosine, as well as adenosine kinase, which phosphorylates adenosine back into AMP (Fig. 1) (12). In the circulation, levels of extracellular adenosine are reported to range from ~0.06 to 0.8 µM (11, 13–15). However, upon infection, adenosine levels in extracellular spaces can increase more than 10-fold (11). Thus, infection changes the concentrations and location (extracellular) of adenosine. Adenosine in the extracellular environment can then act as a signaling molecule by binding to and triggering one of four known G-protein coupled adenosine receptors, named A1, A2A, A2B, and A3 (Fig. 1) (11). Extracellular adenosine binds to and activates these receptors in a dose-dependent manner, where the high-affinity receptor A1 is activated at lower concentrations with EC_50_ < 0.5 µM, intermediate-affinity receptor A2A is activated at EC_50_ > 0.6 µM, and low-affinity A2B receptor (A2BR) is only activated at high levels of adenosine that occur under pathological conditions, with an EC_50_ of 16−64 μM (11). The A3 receptor (A3R) is also reported to, in general, have a lower affinity to adenosine compared to A1 and at times A2A, but affinity varies based on cell type and species tested (16). Activation of the different adenosine receptors can result in opposite effects on cell function that seem to follow the affinity of the receptor. While A1 and A3 receptors are coupled to Gi proteins that inhibit adenylyl cyclase and cAMP production, A2A and A2B receptors are coupled to Gs proteins that activate adenylyl cyclase and increase cAMP (11). A3 and A2B are also coupled to Gq that activates phospholipase C (Fig. 1) (11). These receptors are ubiquitously expressed and can be found on several types of immune cells as well as non-immune cells that shape the outcome of infections (17, 18).

### The role of aging in infection outcomes

By 2050, it is estimated that one in six people will be above the age of 65, totaling two billion worldwide (19–21). With aging comes increased susceptibility to certain serious infections (19–22), such as community-acquired pneumonia (23), urinary tract infections (24), bacteremia (25), infective endocarditis (26), as well as skin/soft tissue infections (27). Our increased susceptibility to these infections as we age presents a serious risk to longevity and quality of life (28). Older adults are not only likely to acquire infections, particularly at mucosal surfaces such as the lungs and urinary tract, but also often present with worse symptoms, suffer from extended recovery times, have higher hospitalization rates, and have an overall worse prognosis (29). Bacterial and viral respiratory infections are particularly prevalent in older adults and are responsible for approximately one-third of all deaths (23). Furthermore, most healthcare dollars spent on hospitalization due to infections are for the treatment of older adults (30–33). Changes in extracellular adenosine production and signaling are known to occur with aging and contribute to the age-related diseases in several organs such as muscle, skin, bone, brain, heart, and metabolism (34–38). However, the role of this pathway in immunosenescence and the age-driven changes in infection outcomes has not been appreciated until the past decade.

In this review, we discuss the role of the extracellular adenosine pathway in shaping the outcome of infections, focusing on its time and dose-dependent effects, its role in the age-driven susceptibility to certain infections, and the feasibility/challenges of therapeutically targeting this pathway for treatment of infections.

## EXTRACELLULAR ADENOSINE AND INFECTIONS

The extracellular adenosine pathway has been extensively studied in acute inflammation and sterile tissue injury models, including hypoxia and ischemia-reperfusion injury in the absence of infection, and has roles in activation and recruitment of several immune cells, as reviewed elsewhere (39–42). Its role in host defense against infections was not appreciated until recently. In host defense against infection, balanced immune responses are associated with the best infection outcome, as too little/no immune response can allow uncontrolled pathogen replication, vs exacerbated immune activation can result in organ damage and loss of function and may not always clear the pathogen (43). Therefore, balancing pathogen clearance while maintaining organ function is needed for optimal host outcome and can be ideally achieved by rapid acute inflammatory responses that can control microbial numbers but that eventually resolve (6, 43). Adenosine’s role in immunity is traditionally reported as suppressive since the production of adenosine reduces levels of extracellular proinflammatory ATP, and the activation of the lower-affinity receptors A2A and A2B suppresses the activity of innate and adaptive immune cells as detailed in the sections below (44). However, extracellular adenosine is not always suppressive, but rather acts as a master regulator of inflammation, where its role in infection outcomes generally follows the principle that a little bit of damage is good, as it activates immune responses, while too much is detrimental (42, 44). In fact, low levels of this damage-associated molecule trigger the high-affinity A1 adenosine receptor that activates immune responses as detailed in the sections below and plays a protective role against infections (45–47). In contrast, continued damage and the production of higher levels of adenosine activate A2A and A2B receptors, which impairs pathogen clearance but also activates resolution of inflammation, which is tissue protective (42, 44, 48–50). Adenosine-producing enzymes and receptors have been reported to shape host interaction with bacteria, viruses, fungi, and parasites, as we discuss below (Table 1).

**TABLE 1:** Time-dependent roles of the extracellular adenosine pathway

Molecule	Time	Infection model	Role	Outcome	References
CD39	Early	*Pseudomonas aeruginosa*	Overexpression on airway epithelium improves bacterial control	Protective	51
	Chronic	HIV/*Mycobacterium tuberculosis*	Expressed on human regulatory T cells Inhibits CD4^+^ T cells Increased expression on CD8^+^ T cells associated with increased mortality in HIV/*M. tuberculosis* co-infected individuals	Detrimental	52 – 54
CD73	Early (hours)	*Streptococcus pneumoniae*	Required for polymorphonuclear cell (PMN) antibacterial function Essential for bacterial clearance	Protective	55, 56
	Chronic- lung	*M. tuberculosis*	Controls PMN recruitment and inflammatory cytokines No effect on bacterial clearance	Tissue protective with varying effect on pathogen clearance	57
	Chronic- gut	*Salmonella*, *Helicobacter*, and *Toxoplasma*	Controls inflammation Impairs pathogen control		58, 59 60
A1 receptor (A1R)	Early (within 6 hours)	*S. pneumoniae*	Required for PMN recruitment and antimicrobial activity Essential for bacterial clearance	Protective	45 – 47
	Later (after 12 hours)	*Yersinia pestis* Sepsis	Inhibition later enhances host survival	Detrimental	61, 62
	Late (after 6 days)	Influenza A infection/sepsis	Required for PMN recruitment Enhances pulmonary damage	Detrimental	62, 63
A2A receptor (A2AR)	Early	*Clostridium difficile/Helicobacter*	Activation reduces inflammation and enhances host survival	Protective	64 – 67
	Later (8−24 hours)	*Escherichia coli/Candida albicans*	Activation later reduces inflammation and enhances pathogen control and host survival	Protective or detrimental- infection dependent	68, 69
	Later	*S. pneumoniae*	Inhibition early has no effect vs later impairs host survival		45
	Late (after day 7)	Autoimmune uveitis/ SARS-CoV-2	Inhibition early has no effect vs later suppresses Th17 and enhances Th1 Later inhibition reduces symptoms in autoimmune uveitis	Promotes tissue injury	70, 71
	Time not tested	*Staphylococcus aureus/Streptococcus suis/Porphyromonas gingivalis*	Impairs PMN antimicrobial function Enhances bacterial replication	Detrimental—infection dependent	72 – 74
A2BR	Early/throughout-lung	*S. pneumoniae and Klebsiella pneumoniae*	Expression impairs PMN antimicrobial function and pathogen clearance	Detrimental	48, 49
	Early/throughout-gut	*C. difficile* toxins/*Helicobacter pylori*	Inhibition reduces inflammation and increases host survival without affecting bacterial numbers	Detrimental	75, 76
	Early/throughout-gut	Sepsis	Opposing findings	Detrimental/context dependent	77, 78
	Independent of time-parasitic infections	*Leishmania donovani/Leishmania amazonensis*	Impairs monocyte and dendritic cell functions Inhibition reduces lesion size and parasite numbers		79 – 81
		Helminth infection	Required for ILC2 responses		82
A3R	Early/throughout	Sepsis	Required for control of bacterial numbers and control of systemic inflammation	Protective (very few studies)	83

### CD39 and CD73

The extracellular enzymes CD39 and CD73 sequentially dephosphorylate ATP into adenosine. These enzymes have been well studied in cancer (84) and inflammatory diseases (85), where they are reported to have anti-inflammatory and T cell suppressive roles. Human regulatory T cells (Tregs) can express either CD73 (58) or CD39 (86), and in mice, Tregs can express both (87). Extracellular adenosine production by Tregs is one of the mechanisms by which they can suppress T cells. Extracellular adenosine produced by Tregs acts on A2A or A2B receptors expressed on T cells to blunt T cell effector functions like IFNγ production and cytotoxicity, as well as proliferation (87). In infection, these enzymes suppress inflammatory responses and have both protective and detrimental roles depending on the context (Table 1).

In pulmonary infections, CD73 and CD39 protect the host by dampening inflammation and/or affecting pathogen clearance (18). In humans, COVID-19 patients with more severe disease had lower levels of CD73 on circulating immune cells and lower adenosine levels in the circulation as compared to healthy controls (88, 89). In a *Pseudomonas aeruginosa* acute infection model, overexpression of CD39 on the airway epithelium increased adenosine levels in alveolar lavage fluid and resulted in better control of bacterial numbers in the lungs (51). In pneumococcal pneumonia, CD73^−/−^ mice suffered from exacerbated pulmonary inflammation and higher polymorphonuclear cell (PMN) recruitment to the lung but defective PMN antibacterial function (55, 56). CD73^−/−^ PMNs failed to produce extracellular adenosine and kill pneumococci *ex vivo*, a defect that was reversed by exogenous supplementation with adenosine (47, 55). As a result, the CD73^−/−^ mice failed to control *Streptococcus pneumoniae* (pneumococcus) numbers early in infection and succumbed to the disease at a significantly higher rate compared to wild-type controls (56). Similarly, in *Mycobacterium tuberculosis* infection, CD73^−/−^ mice had higher PMN numbers and inflammatory cytokine levels in the lungs but displayed no change in the ability of their macrophages to kill the bacteria and had similar bacterial loads in the lung as wild-type controls between days 21 and 84 following challenge (57). Long-term treatment of mice with caffeine, which competes with adenosine for receptor binding, enhanced IFNγ production by pulmonary T cells and extended host survival without affecting *M. tuberculosis* numbers (90). These findings suggest that in acute bacterial infections, adenosine production by CD39 and CD73 in the lungs is needed to blunt pulmonary inflammation and enhance bacterial clearance, but in chronic infection, sustained adenosine signaling may be detrimental to the host. In fact, in HIV-infected individuals, adenosine production and CD39 expression by regulatory T cells result in inhibition of CD4^+^ T cells via the A2A receptor (A2AR) (52, 53). CD39 expression correlated with higher HIV titer, lower circulating CD4^+^ T cells, and increased death (52). Individuals with single-nucleotide polymorphisms (SNPs) that reduce CD39 expression were associated with slower AIDS progression (52) vs higher levels of CD39-expressing CD8^+^ T cells were associated with increased mortality in HIV/*M. tuberculosis* co-infected individuals (54).

In intestinal infections with *Salmonella* (59) or *Helicobacter* (58), CD73^−/−^ mice had enhanced bacterial clearance; however, these mice suffered from significantly higher levels of inflammatory cytokines. In *Helicobacter* infection, the absence of CD73 was linked to symptoms of gastritis as CD73 expression on regulatory T cells was required to control inflammation (58). Similarly, reduced levels of CD73 expression and subsequent adenosine levels in the gut in response to oral inoculation with *Toxoplasma gondii* resulted in inflammation (60). Activation of signaling via A2A and A2B receptors lowered inflammation, restored intestinal barrier integrity, and resulted in lower parasite burden during the acute phase of toxoplasmosis (60). In contrast, during the chronic phases of the disease, adenosine production by CD73 was needed for efficient differentiation of the parasite into bradyzoites in the brain, but this was independent of inflammation in the brain or signaling via A1 or A2A receptors and was proposed to be a direct effect of adenosine on *Toxoplasma gondii* that needs to scavenge purines from the host as it cannot make its own (91). Therefore, in the context of intestinal infection, adenosine production by CD73, while it may have varying effects on pathogen numbers, is consistently tissue protective. In conclusion, while CD73 and CD39 are tissue protective in acute infection settings, sustained adenosine production, due to its inhibitory activity and depending on the amounts produced, is detrimental for the control of chronic infections (Table 1).

### A1 receptor

A1 receptor (A1R) is a high-affinity receptor that is activated by low levels of adenosine. This receptor activates innate immune cells and has been consistently reported to enhance inflammation (42). It is ubiquitously expressed and exerts control of infection via its actions on both immune and non-immune cells (46, 47). During *S. pneumoniae* pulmonary infection, A1 receptor signaling was required for host survival and control of bacterial numbers in the lungs and blood early in infection (45–47). A1R controlled neutrophilic responses, where it was required for upregulation of the chemokine receptor CXCR2 on neutrophils and for the ability of these cells to migrate across the endothelial barrier into the lungs within the first 6 hours of infection (45). It was also required for neutrophil antimicrobial activity against *S. pneumoniae* (47). A1R was also highly expressed in the lungs on pulmonary epithelial cells and prevented bacterial binding to these cells by downregulating the expression of platelet-activating factor receptor, which is used by *S. pneumoniae* as an adhesin (46). In line with findings during bacterial infection, A1R expression on neutrophils was required for their ability to influx into the lungs in a murine model of influenza A infection (IAV) (63). However, unlike pneumococcal pneumonia, A1 receptor signaling did not affect viral burdens in the lungs and was detrimental for host outcome during IAV infection (63). Genetic deletion or pharmacological inhibition of A1R led to lower pulmonary damage and improvement of pulmonary function (63, 92, 93). Optimal lung function occurred when A1 was absent from immune and non-immune cells (63). The detrimental effects of A1R were not observed early in IAV infection, but rather later by day 6 (63). This suggests that the kinetics of A1R signaling play a key role in host defense, in which early activation of A1R is host-protective but sustained activation is not. This could be due to A1 receptor’s effect on immune activation, as overt inflammation is detrimental for infection outcome. Therefore, continuous activation of the A1R can become damaging for the host.

In fact, data support a time-dependent role for this receptor, where inhibition later in infection is protective (61, 62). In a sepsis model using cecal ligation and double puncture (CLP), genetic deletion or pharmacological inhibition of A1R signaling resulted in a decline in liver and kidney function and decreased survival in mice (61). In contrast, treatment with an A1R inhibitor in combination with antibiotic treatment 12 hours after induction of sepsis led to enhanced survival and lower signs of kidney damage (62). Similarly, in a *Yersinia pestis* pulmonary infection model, inhibition of A1R in combination with antibiotic therapy starting 72 hours following challenge significantly improved survival of infected rats (94). In summary, findings suggest that activation of A1R early in infection is beneficial for the outcome of host-pathogen interactions, but its sustained activation is not (Table 1).

### A2A receptor

A2A receptor has lower affinity to adenosine compared to A1 but is still activated by lower levels of adenosine (11). A2AR has been reported to have anti-inflammatory effects and to be either protective or detrimental depending on the infection type and the timing of activation. In gastrointestinal infections, A2AR plays a beneficial role where its activation protects hosts against *Clostridium difficile* toxin in several animal models by reducing intestinal cell death and enhancing host survival (64–67). It similarly protected against gastric injury in *Helicobacter* infections (95). This receptor also has a clear time-dependent role in infection outcome. In a *S. pneumoniae* murine infection model, inhibition of A2AR signaling prior to bacterial pulmonary challenge had no effect on host survival; however, inhibition 18 hours post-infection significantly decreased survival, suggesting that A2AR plays a protective role later in infection (45). The infection itself resulted in dynamic changes in all adenosine receptors, where expression of A1, A2A, A2B, and A3 on circulating and pulmonary PMNs decreased within the first 6 hours of infection, followed by recovery to basal levels by 48 hours (45). Similarly, in an *Escherichia coli* peritonitis model of infection, expression of the different adenosine receptors changed over time, where A1R peaked first at 12 hours post-challenge followed by a decrease, A2AR peaked second at 24 hours then decreased by 48 hours, and A2BR did not increase until after 24 hours, suggesting that these receptors play a time-dependent role in infection (96). In systemic infection with *E. coli*, pharmacological activation of A2AR 8 hours post-challenge, in combination with antibiotics, significantly enhanced survival of murine hosts (68). Similarly, in *Candida albicans* systemic infection, activation of A2AR 1 day following infection reduced pathogen numbers and boosted host survival by reducing inflammation in the kidneys and promoting an anti-inflammatory phenotype in tissue macrophages (69). In a model of autoimmune uveitis, inhibition of A2AR at day 1 post-immunization had no effect on disease progression, while inhibition at day 7 was tissue protective and mediated its effects via suppression of γδ T cells and Th17 responses (70). Inhibition of A2AR similarly suppressed Th17 and enhanced Th1 responses and was protective in a murine model of SARS-CoV-2 infection (71). These and other findings (97, 98) suggest that in inflammatory settings, A2AR activation on CD4^+^ T cells shifts the Th1/Th17 balance toward Th17. Several bacteria have evolved to activate A2AR signaling to evade clearance. Both *Staphylococcus aureus* (72) and *Streptococcus suis* (73) activate A2AR signaling on PMNs to evade intracellular killing by impairing antimicrobial activities such as degranulation and reactive oxygen species (ROS) production. Similarly, activation of A2AR signaling on gingival epithelial cells resulted in enhanced intracellular *Porphyromonas gingivalis* bacterial replication (74). In summary, these studies suggest that the role of A2AR is infection dependent, but the timing of activation also has a crucial effect on its activity (Table 1).

### A2B receptor

A2B receptor is a low-affinity receptor that is activated by high levels of adenosine that accumulate under pathological conditions. This receptor has been reported in general to impair host defense against bacterial infections and to have both protective and detrimental roles against parasitic infections. During pulmonary infection with the gram-negative bacteria *Klebsiella pneumoniae*, A2BR^−/−^ mice had lower bacterial burdens and survived the infection better than wild-type controls (49). Similarly, A2BR^−/−^ mice were more resistant to lung infection with *S. pneumoniae* (48). In both instances of bacterial pneumonia, A2BR impaired the antimicrobial function of PMNs but via different mechanisms. In response to *K. pneumoniae* infection, A2BR blunted the formation of neutrophil extracellular traps by PMNs but did not have any effect on bacterial uptake or production of total ROS (49). In response to *S. pneumoniae* infection, A2BR inhibited mitochondrial ROS production in both murine and human PMNs (48). Mitochondrial, but not NADPH oxidase ROS, were required for efficient pneumococcal killing by PMNs and for the increased resistance of A2BR^−/−^ mice to systemic infection (48). PMN recruitment and pulmonary pathology were not affected in both models of pneumonia (48, 49); therefore, A2BR impairs host defense by blunting antibacterial immunity.

In intestinal bacterial infections, the presence of A2BR exacerbated inflammation without affecting bacterial numbers. In *C. difficile* murine and rabbit infection models, inhibition or genetic deletion of A2BR reduced *C. difficile* toxin A- and B-mediated intestinal pathology and inflammation as well as diarrhea and enhanced host survival without affecting bacterial shedding (75). Similarly, in a rat model of *Helicobacter pylori* gastric ulcers, inhibition of A2BR reduced inflammation and ulcer area (76). In CLP sepsis models, there are opposing data regarding the role of A2BR. In some studies, inhibition or deletion of A2BR was reported to improve host outcome and survival by reducing systemic bacterial numbers and overall inflammation (77), while in other studies, the reverse was reported (78). This could be driven by differences in the composition of the microbiome, which is known to vary across institutes and directly alter host responses.

In parasitic infections, A2BR plays specific roles depending on the type of parasite. For example, A2BR plays a detrimental role in leishmaniasis. In *Leishmania donovani* patients, elevated expression of A2BR on monocytes correlated with worse disease score (99). *Leishmania amazonensis* inhibited dendritic cell function by upregulating expression of A2BR on the cell surface, and blocking A2BR signaling in mice ameliorated skin lesion size and parasite numbers (79–81). In contrast, during helminth infection, A2BR was needed for the production of IL-33 and the induction of type 2 innate and adaptive immunity, and its inhibition resulted in lower parasite elimination (82). In summary, findings suggest that the role of A2BR depends on the type of infection, but overall, it blunts pro-inflammatory responses during infection (Table 1).

A2A and A2B receptors are both Gs coupled and activate adenylyl cyclase but do not always play redundant roles in infection. In some instances, they have similar roles, such as in *Haemophilus influenzae* infection, where inhibition of either receptor improved barrier integrity in an endothelial-pericyte coculture model of the blood-brain barrier (100). In contrast, in CLP sepsis studies (performed by the same group), they play opposing roles where genetic deletion of A2AR (101) improves host survival, while deletion of A2BR does the reverse (78). In some infections, only one of the receptors is involved in the host outcome, and that could be linked to expression levels. For example, while A2A plays no role in pneumococcal pneumonia early in infection (45), A2B does (48). This could be due to expression levels where A2BR is highly expressed on epithelial cells (46) under hypoxic conditions, such as during infection. During hypoxia, hypoxia-inducible factor 1α is upregulated and binds to its binding site within A2B receptor’s promoter region, leading to increased receptor expression (102, 103). Similarly, genetic deletion of A2BR but not A2AR protects against death of enteric glial cells following intoxication with *C. difficile*, and intoxication increases A2AR levels but decreases A2BR levels (64–66). *L. amazonensis* recruits A2BR but not A2AR on the dendritic cell (DC) cell surface to inhibit immune activation (79). Activation of A2AR but not A2BR is protective against *Mycobacterium leprae* infection of Schwann cells, and that could again be due to differences in expression where infection downregulates A2A but not A2BR levels (104). These findings suggest that factors within the tissues other than adenosine levels alone, including manipulation of receptor expression by pathogens, can shape the roles for the different adenosine receptors in infection.

### A3 receptor

The role of the A3 receptor is the least studied in infection settings due to its more recent discovery compared to the other receptors (discovered in 1992) (105). It is expressed on immune cells such as PMNs and plays a key role in their chemotaxis and recruitment to inflamed tissue (106–108). Its role has been primarily tested in CLP sepsis models where A3R^−/−^ mice consistently show higher bacterial burdens systemically following intestinal injury, and while they have less inflammation in the lungs, they display higher inflammation in the kidneys and have lower survival rates over time (83). More studies are needed to test if A3R has a role in other infection settings.

### Manipulation by pathogens

Apart from alteration of receptor expression levels as discussed above, many pathogens have also evolved to affect adenosine production and receptor activation. *Toxoplasma gondii*, for example, uses host-derived adenosine production by CD73 for efficient differentiation in the brain (91). Several bacterial species express their own 5’-ectonucleotidases and produce adenosine to manipulate host responses. *Legionella pneumophila* expresses multiple such enzymes with similarities to host CD39 that dephosphorylate ATP and ADP (109, 110). Deletion of these enzymes, called Lpg0971 or Lpg 1905, resulted in reduced intracellular bacterial replication in macrophages and amoeba and lower virulence in a mouse model of pneumonia (109, 110). *S. aureus* expresses a cell wall-anchored adenosine synthase (AdsA) that can dephosphorylate both ADP and AMP into adenosine (111). AdsA was required for the ability of *S. aureus* to survive intracellularly in human neutrophils and for infection of tissues in a murine model of systemic infection (111). Several streptococcal species also express ectonucleotidases. *S. suis* expresses the cell surface enzyme adenosine synthase (Ssads) that hydrolyzes AMP to adenosine (73). This enzyme is required for bacterial survival in human blood and PMNs, as it suppresses PMN degranulation and antimicrobial activity by activating the A2AR (73). Ssads is also used by the bacteria to cross the blood-brain barrier, where adenosine production by the bacteria activates A1R signaling, allowing bacterial translocation across the endothelial barrier (112). Similarly, *Streptococcus agalactiae* expresses NudP, a cell surface enzyme that dephosphorylates ADP and AMP to adenosine (113). NudP was required for bacterial survival in human blood and virulence in a systemic infection model in mice (113). *Streptococcus sanguinis* also expresses an ectonucleotidase that can hydrolyze ATP to adenosine, which was required for bacterial virulence and infection in a model of endocarditis (114). While *S. pneumoniae* has not been shown to express a 5’-ectonuclease (113), these bacteria can interfere with adenosine production via removal of surface CD73 on host cells (55), which is necessary for host survival (46, 47, 56). The mechanisms by which they do so are under investigation. These findings indicate that pathogens have an active role in modulating the extracellular adenosine pathway, which could explain the infection-specific role these receptors play in host defense.

Taken all together, these studies suggest that the extracellular adenosine damage response signaling pathway acts as a master regulator of host response to infections, where low levels of damage activate the proinflammatory/immune-activating A1R and initiate antimicrobial defense pathways (Fig. 1; Table 1). However, prolonged activation is detrimental to the host, and eventual higher buildup of adenosine allows activation of A2A, whose role in infection comes in a bit later and, although it can dampen antimicrobial activity of immune cells, can also be tissue protective. When high levels of adenosine build up, A2BR is activated and plays a role in suppressing immune responses and dampening inflammation (Fig. 1; Table 1). This could be a built-in mechanism for the host to prevent excessive tissue damage due to inflammation. Therefore, if adenosine builds up too rapidly in the extracellular space, that could be detrimental for control of infection, but if it accumulates slower over time, activation of A2A and/or A2B may then be beneficial for resolving immune activation. Importantly, there is cross talk between the extracellular adenosine pathway and infectious organisms that have evolved ways to manipulate this pathway for their benefit, supporting its importance in host defense.

## EXTRACELLULAR ADENOSINE AND IMMUNOSENESCENCE

Aging is associated with genomic, molecular, cellular, and systemic hallmarks that accumulate with time and drive the aging process (19, 115). There are currently 14 hallmarks proposed by Lopez-Otin and colleagues (19, 115) that are divided into primary hallmarks that drive the aging process (genomic stability, telomere shortening, epigenetic changes, loss of proteostasis, and reduced autophagy), antagonistic hallmarks mounted to the primary changes (altered nutrient sensing, aberrant mitochondrial function, and cellular senescence), and integrative hallmarks that result in the aging phenotype, driven by the primary and secondary hallmarks (chronic inflammation, stem cell exhaustion, dysbiosis, changes in the extracellular matrix, and altered intracellular communications). These changes have a serious impact on immune responses and the ability of the host to fight and recover from certain infections (116, 117). Immunosenescence, defined as the decline in immune cell function with age, has been described for most innate and adaptive immune cells and results in impaired ability to kill certain infecting organisms (118). This is in part driven by inflammaging, the low-grade chronic inflammation (118) that accompanies aging and blunts the ability of immune cells to acutely respond to infection. One source of inflammaging is senescent cells. Cellular senescence is defined as a state of stable cell cycle arrest in response to a variety of stressors whereby cells remain viable and metabolically active but lose the ability to divide, become resistant to apoptosis, and develop altered responses including senescence-associated secretory phenotype characterized by the production of inflammatory mediators such as TNF-α, IL-1, and IL-6 (119). Aging is also associated with exacerbated tissue and organ damage in response to infections, particularly in the lungs, due to the reduced/delayed ability to resolve inflammation (120).

Changes in the extracellular adenosine pathway have been proposed to contribute to the aging process (36). Age-driven changes in extracellular adenosine production and signaling have been described in several organs/systems, and the extracellular adenosine pathway plays a role in age-related pathologies of many organs, including the brain, heart, bone, skin, muscle, and joints, and also contributes to the altered metabolism seen with age (34–38). Targeting A2A receptor signaling in particular has been described to reverse some of the hallmarks of aging in some systems, including preventing cellular senescence, as recently reviewed in detail by Rabbani and colleagues (36). Recent work in the past decade has also highlighted the role of the extracellular adenosine pathway in immunosenescence and the age-driven changes in infection outcomes, as we describe below.

### Pathway expression and immune cell function

The levels of circulating adenosine, as well as the enzymes that make and break adenosine, along with the various adenosine receptors on immune cells, have been reported to change with age (47, 121–128) (Fig. 1).

In mice, at baseline, in the absence of infection, levels of adenosine in the circulation inversely correlated with age, and aging was associated with reduced plasma ATP hydrolysis to adenosine (121). Neutrophils can be a major source of adenosine (129), and with age, there is lower CD73 expression and production of adenosine by bone-marrow-derived neutrophils in murine models (47). Lower adenosine production by murine PMNs was linked to a decline in bacterial killing, and supplementing adenosine back rescued the ability of PMNs from aged mice to kill *S. pneumoniae* (47). In humans, PMNs may express CD73 intracellularly (130) rather than on their surface (129), and CD73 activity is required for the antimicrobial function of PMNs in young donors (55), but age-driven changes of CD73 expression in human PMNs have not been examined. There are well-known age-associated changes in CD73 and CD39 on T cells. Older adults (who were less than 100 years old) expressed higher levels of CD73 and CD39 mRNA in total peripheral blood cells (122). In *in vitro* cultures, T cells from older adults released more adenosine compared to younger controls (123). When examining CD4^+^ and CD8^+^ T cells, CD73 expression declined with aging (124, 125) and denoted a subset of memory cells that responded to stimulation with viral peptides (124). In contrast, CD39 expression on CD4^+^ T cells increased with aging on activated effector T cells, and these cells were unable to promote B cell differentiation to plasmablasts (126). Participants with SNPs that lowered the expression of CD39 had better responses to Varicella Zoster and Flu vaccines (126). CD39 is also expressed on regulatory T cells, and one of the ways these cells mediate immune suppression is via adenosine production (86, 131). These findings suggest that age-driven changes in adenosine-producing enzymes CD73 and CD39 on T cells may be linked with the decline in vaccine responses with aging; however, more functional studies testing if inhibiting adenosine production or signaling can improve vaccine responses are needed.

Adenosine deaminase, by degrading adenosine, reduces replicative senescence in CD8^+^ T cells (132), and its activity was reported to be lower in peripheral blood lymphocytes of older human participants (133). Changes in adenosine receptor levels on immune cells have also been reported with age. In humans, mRNA levels of the A2A receptor on circulating lymphocytes increased with aging (122). Importantly, pharmacologically inhibiting A2AR reversed some of the age-driven defects in T cell responses to acute stimuli, including expression of the costimulatory marker CD28 on CD3-activated T cells and chemotaxis in response to CCL21 (123).

### Response to infection

Aging is associated with an increased susceptibility to certain types of infections, including pulmonary and invasive pneumococcal disease caused by *S. pneumoniae* (134), SARS-CoV-2 (135), *Listeria monocytogenes* meningitis, and intestinal infections (136), as well as invasive candidiasis (137). Age-driven changes in the extracellular adenosine pathway in response to these infections have been reported. Both enzyme and receptor levels change with aging in response to infection at the tissue level and on immune cells, with most studies reporting changes in T cells and PMNs. In *L. monocytogenes* intestinal infection, aged mice were unable to control bacterial numbers as efficiently as young controls and had elevated damage in peripheral tissues (127). This was linked to higher IL-10 and IL-17 but lower IFNγ production by T cells and higher expression of CD39 and CD73 on Tregs (127). COVID-19 patients with more severe disease, who were overall older than the healthier cohorts, had higher amounts of CD39^+^ CD4^+^ T cells and lower amounts of CD73^+^ CD8^+^ T cells as well as lower amounts of adenosine in the circulation compared to those with less severe disease and healthy controls (88, 89). In an oral infection model of *Candida albicans*, aged mice were unable to control pathogen numbers and failed to upregulate expression of A2AR in intestinal tissues, unlike young controls that upregulated A2AR and suffered less tissue damage in response to infection (128). In an air pouch lipopolysaccharide (LPS) inflammation model, aging was associated with decreased inflammatory cytokine production and lower PMN influx and activation (138). In the absence of A2AR, these changes were apparent earlier on in mid-aged mice, suggesting that A2AR signaling protected against these age-driven alterations in immune responses (138). In *S. pneumoniae* pulmonary infection, aged mice suffered from higher bacterial numbers, exacerbated pulmonary inflammation, and succumbed to the infection at a significantly higher rate than young controls (139). Expression of CD73 in the lungs and expression of all four adenosine receptors on circulating and pulmonary PMNs decreased significantly more in aged mice compared to young controls (45).

Studies also suggest that adenosine signaling is dysregulated with aging. T cells from older adults were reported to be less responsive to adenosine stimulation in several studies (123, 133). In *S. pneumoniae* infection, signaling via the A1R on PMNs was required for transendothelial migration and early recruitment to the lungs (45) as well as their antimicrobial activity (47). However, aged mice had a delay in early PMN pulmonary recruitment, and activation of A1 receptor signaling reversed the age-driven decline in the ability of PMNs to migrate across the endothelium into the lungs and boosted PMN antimicrobial function (45). A1R agonism reversed the susceptibility of aging to infection, and activation of A1R at the time of infection resulted in lower *S. pneumoniae* bacterial burden and enhanced survival following pulmonary infection in aged mice (46). Adenosine production was also significantly higher in aged mice compared to young controls later in *S. pneumoniae* infection (45), suggesting that the lower-affinity A2BR, which is detrimental to host defense against these bacteria, may be activated. However, inhibition of A2A or A2B receptors in aged mice was unable to boost host defense against *S. pneumoniae* (45, 140). Overall, these findings demonstrate that there are age-driven changes in the extracellular adenosine pathway in response to infection that could play a role in the increased susceptibility to infections seen with age. The mechanisms behind these changes and whether they are driven by hallmarks such as inflammaging are an open area of research. In summary, the above studies support the hypothesis that changes in the extracellular adenosine damage response signaling pathway with aging feed into the aging hallmark of altered intercellular communication and contribute to the susceptibility of aged hosts to certain infections.

## EXTRACELLULAR ADENOSINE IN CLINICAL AND TRANSLATIONAL STUDIES

Most studies examining the role of the extracellular adenosine pathway in infections have been preclinical in animal models. However, few groups have confirmed findings in preclinical infection models using cells from human participants, while others have also tracked the expression of the different adenosine pathway components in human participants or looked for genetic associations between this pathway and risks of infections, as discussed below.

### Expression studies

Age-driven changes in the expression of adenosine-producing enzymes and the role of adenosine signaling in the decline in T cell function have been described in human participants as described in the section above (124–126). Similarly, at the other extremes of age, infants had higher adenosine deaminase activity than adolescent participants, and this correlated with reduced ability to phagocytose bacteria (141). The inhibitory effects of A2B (48) and A2A (72) receptors on the ability of PMNs to kill *S. pneumoniae* and *S. aureus*, respectively, have been confirmed in cells from human donors. Few studies have also tracked expression of the adenosine pathway during infections and inflammation. In COVID-19 patients, lower CD73 expression and adenosine production in the circulation were associated with more severe disease (88, 89). In HIV infection, the reverse was true, where adenosine production by regulatory T cells resulted in immune suppression, and lower CD39 expression was associated with improved prognosis and slower progression to AIDS (52). In septic shock patients, adenosine levels were significantly higher in the circulation compared to healthy controls, and *in vitro* activation of A2AR on circulating patient PMNs or treatment with high concentrations of adenosine reduced the elevated ROS production in response to TNF-α and N-formylmethionyl-leucyl-phenylalanine (fMLP) seen in these patients (142). Similarly, another study reported an increase in adenosine in the circulation of sepsis patients and participants treated with LPS to experimentally induce inflammation (143). LPS-treated participants displayed decreased mRNA expression of ADA, while CD73 was increased on circulating lymphocytes. LPS-treated participants also had decreased mRNA levels of the proinflammatory A1 and A3 receptors vs an increase in the expression of A2A and A2B (143). Similar receptor changes were reported in patients with chronic periodontitis, where expression of A1 and A3 was lower, while expression of A2A and A2B was higher in the gingival tissues of patients compared to healthy controls (144). Together, these findings suggest that high levels of circulating adenosine may act as an anti-inflammatory signal in patients with inflammatory diseases. Overall, the extracellular adenosine pathway undergoes dynamic changes in response to infection and inflammation in humans, and more studies are needed to understand the mechanisms behind these changes and whether these changes play a role in host outcome following infection.

### Genetic studies

SNPs in all four extracellular adenosine receptors and enzymes that make or convert adenosine have been described in the human population and have been associated with several inflammatory diseases (145–149). However, only a handful of studies examining associations between SNPs in the components of the extracellular adenosine pathway and infections have been done. A study with a small cohort of SARS-CoV-2 patients in Portugal found no association between SNPs in A2AR and the severity of disease (150). On the other hand, two SNPs (rs9624472 and rs5751876) in A2AR were associated with increased risk of malaria caused by *Plasmodium falciparum* (151). Another SNP (rs2298383) that reduces expression of A2AR was found to be associated with increased risk of multiple chemical sensitivity, an inflammatory disease that renders patients less tolerant to chemicals encountered in daily life (152). Several SNPs in the adenosine degrading enzyme ADA (rs121908715 and rs121908723) were associated with a significantly higher risk of tuberculous pericarditis, a severe disease that develops in patients infected with *M. tuberculosis* in a Chinese cohort (153). A SNP (rs17602729) in the enzyme AMP deaminase, which results in loss of function and accumulation of adenosine due to increased AMP availability, was associated with increased occurrence of community-acquired pneumonia that was accompanied by sepsis in a cohort in Greece (154). SNPs (rs11188513) that reduced CD39 expression were linked to slower progression to AIDS in HIV patients (52). These few studies clearly show a link between polymorphisms in several extracellular adenosine pathway components and risks of developing certain infections. However, given the preclinical data in animal models reviewed above, more population studies are needed, particularly looking at associations of infections at mucosal sites such as the lungs and intestines.

### Drugs targeting the extracellular adenosine pathway

There are several drugs that target the extracellular adenosine signaling pathway directly. These include agonists and antagonists specific for the A2A, A2B, and A3 receptors as well as inhibitors against ADA and CD73 that are in clinical trials for the treatment of a wide range of conditions, including inflammatory diseases, as extensively reviewed elsewhere (12, 155, 156). The only direct adenosine-based drugs approved by the Food and Drug Administration (FDA) are adenosine, which was approved in 1989 for treatment of tachyarrhythmias but was recently withdrawn due to side effects, and two A2AR targeting drugs (12). One is Regadenoson, an A2AR agonist approved for use in myocardial perfusion imaging (157, 158), and Istradefylline, an A2AR antagonist approved for use in combination with other therapies for the management of Parkinson’s disease (159). Regarding the treatment of infections, drugs that target the adenosine pathway have been mainly tested in clinical trials for the treatment of viral infections. A phase II ARCTIC trial (NCT04588441) testing aerosolized inhaled adenosine treatment in patients with acute respiratory distress syndrome caused by COVID-19 is ongoing. A phase 1b trial (NCT04606069) using the A2A agonist Regadenoson for treatment of COVID-19 patients found that the infused drug was safe and reduced inflammatory cytokines in the circulation (160). Other drugs that have adenosine pathway modulating effects as one part of their mechanisms of action have also been approved for several diseases, and in the context of infections, the antiviral drug Remdesivir was shown to mediate host protection against SARS-CoV-2 in part through its metabolite acting as an A2AR antagonist (71). Ribavirin, an antiviral drug that has activities against the hepatitis C virus (161) and is in clinical trials (NCT06744777) for the treatment of viral hemorrhagic fevers, indirectly increases adenosine levels and activates A1R (12). None of the trials target older adults specifically. In preclinical models, activating A1R boosted the resistance of aged mice to pulmonary pneumococcal infection (46). However, while full A1 receptor agonists have reached clinical trials for the treatment of several diseases, they are reported to have detrimental cardiovascular effects *in vivo* and are not well tolerated (155, 162). Therefore, moving forward, the design of partial agonists or prodrugs activated locally at the sites of infection could be potential strategies for interventions against pneumococcal infections in older adults (163).

## SUMMARY AND FUTURE PERSPECTIVES

In summary, the production and signaling of adenosine in the extracellular environment are a damage response mechanism that plays a key role in infection outcomes. Extracellular adenosine has a dose- and time-dependent effect on the ability of hosts to mount antimicrobial responses and control pathogen numbers, as well as to control tissue damage resulting from immune activation following infection. Several infectious organisms actively alter extracellular adenosine production and the levels of the different adenosine receptors, and the ability to do so is important for their pathogenicity and virulence. Aging is accompanied by changes in expression of the extracellular adenosine pathway components as well as changes in receptor signaling in tissues and on immune cells in response to several infections. This suggests that damage response signaling via this pathway is altered during aging and may drive detrimental host outcomes following certain infections. In fact, pharmacologically targeting extracellular adenosine signaling reverses the age-driven changes in neutrophil and T cell responses in several models and boosts host resistance to pneumococcal pneumonia in preclinical settings (45, 47, 124–126). However, the role of the extracellular adenosine pathway in immunosenescence is just starting to be explored. Understanding the mechanisms by which changes in extracellular adenosine production and signaling with age control host defense against infections is needed, as are more studies testing if targeting the extracellular adenosine pathway therapeutically can boost host resistance to a wider range of age-associated infections.

## References

[1] Chen H, Matsumoto H, Horita N, Hara Y, Kobayashi N, Kaneko T. 2021. Prognostic factors for mortality in invasive pneumococcal disease in adult: a system review and meta-analysis. Sci Rep 11:11865. doi:10.1038/s41598-021-91234-y 34088948 PMC8178309

[2] Kradin RL, Digumarthy S. 2017. The pathology of pulmonary bacterial infection. Semin Diagn Pathol 34:498–509. doi:10.1053/j.semdp.2017.06.001 28655479

[3] Novak R, Tuomanen E. 1999. Pathogenesis of pneumococcal pneumonia. Semin Respir Infect 14:209–217. 10501308

[4] Ayres JS, Schneider DS. 2012. Tolerance of Infections. Annu Rev Immunol 30:271–294. doi:10.1146/annurev-immunol-020711-075030 22224770

[5] Promislow D, Anderson RM, Scheffer M, Crespi B, DeGregori J, Harris K, Horowitz BN, Levine ME, Riolo MA, Schneider DS, Spencer SL, Valenzano DR, Hochberg ME. 2022. Resilience integrates concepts in aging research. iScience 25:104199. doi:10.1016/j.isci.2022.104199 35494229 PMC9044173

[6] Casadevall A, Pirofski L. 2003. The damage-response framework of microbial pathogenesis. Nat Rev Microbiol 1:17–24. doi:10.1038/nrmicro732 15040176 PMC7097162

[7] Ma M, Jiang W, Zhou R. 2024. DAMPs and DAMP-sensing receptors in inflammation and diseases. Immunity 57:752–771. doi:10.1016/j.immuni.2024.03.002 38599169

[8] Land WG. 2015. The role of damage-associated molecular patterns in human diseases : part I - promoting inflammation and immunity. Sultan Qaboos Univ Med J 15:e9–e21. doi:10.18295/2075-0528.1648 25685392 PMC4318613

[9] Roh JS, Sohn DH. 2018. Damage-associated molecular patterns in inflammatory diseases. Immune Netw 18:e27. doi:10.4110/in.2018.18.e27 30181915 PMC6117512

[10] Schaefer L. 2014. Complexity of danger: the diverse nature of damage-associated molecular patterns. J Biol Chem 289:35237–35245. doi:10.1074/jbc.R114.619304 25391648 PMC4271212

[11] Haskó G, Linden J, Cronstein B, Pacher P. 2008. Adenosine receptors: therapeutic aspects for inflammatory and immune diseases. Nat Rev Drug Discov 7:759–770. doi:10.1038/nrd2638 18758473 PMC2568887

[12] Kutryb-Zając B, Kawecka A, Nasadiuk K, Braczko A, Stawarska K, Caiazzo E, Koszałka P, Cicala C. 2023. Drugs targeting adenosine signaling pathways: a current view. Biomed Pharmacother 165:115184. doi:10.1016/j.biopha.2023.115184 37506580

[13] Groppelli A, Brignole M, Chefrour M, Gastaldi M, El Oufir F, Deharo JC, Parati G, Guieu R. 2022. Adenosine concentration in patients with neurally mediated syncope. Front Cardiovasc Med 9:900023. doi:10.3389/fcvm.2022.900023 35800167 PMC9254326

[14] Funaya H, Kitakaze M, Node K, Minamino T, Komamura K, Hori M. 1997. Plasma adenosine levels increase in patients with chronic heart failure. Circulation 95:1363–1365. doi:10.1161/01.CIR.95.6.1363 9118500

[15] Simard T, Jung R, Labinaz A, Faraz MA, Ramirez FD, Di Santo P, Perry-Nguyen D, Pitcher I, Motazedian P, Gaudet C, Rochman R, Marbach J, Boland P, Sarathy K, Alghofaili S, Russo JJ, Couture E, Promislow S, Beanlands RS, Hibbert B. 2019. Evaluation of plasma adenosine as a marker of cardiovascular risk: analytical and biological considerations. J Am Heart Assoc 8:e012228. doi:10.1161/JAHA.119.012228 31379241 PMC6761640

[16] Gao ZG, Auchampach JA, Jacobson KA. 2023. Species dependence of A3 adenosine receptor pharmacology and function. Purinergic Signal 19:523–550. doi:10.1007/s11302-022-09910-1 36538251 PMC9763816

[17] Bhalla M, Simmons SR, Abamonte A, Herring SE, Roggensack SE, Bou Ghanem EN. 2020. Extracellular adenosine signaling reverses the age-driven decline in the ability of neutrophils to kill Streptococcus pneumoniae. Aging Cell 19:e13218. doi:10.1111/acel.13218:e13218 32790148 PMC7576260

[18] Lee JS, Yilmaz Ö. 2018. Unfolding role of a danger molecule adenosine signaling in modulation of microbial infection and host cell response. Int J Mol Sci 19:199. doi:10.3390/ijms19010199 29315226 PMC5796148

[19] López-Otín C, Blasco MA, Partridge L, Serrano M, Kroemer G. 2013. The hallmarks of aging. Cell 153:1194–1217. doi:10.1016/j.cell.2013.05.039 23746838 PMC3836174

[20] Tenchov R, Sasso JM, Wang X, Zhou QA. 2024. Aging hallmarks and progression and age-related diseases: a landscape view of research advancement. ACS Chem Neurosci 15:1–30. doi:10.1021/acschemneuro.3c00531 38095562 PMC10767750

[21] Yoshikawa TT. 2000. Epidemiology and unique aspects of aging and infectious diseases. Clin Infect Dis 30:931–933. doi:10.1086/313792 10880303

[22] Gavazzi G, Krause KH. 2002. Ageing and infection. Lancet Infect Dis 2:659–666. doi:10.1016/s1473-3099(02)00437-1 12409046

[23] Safiri S, Mahmoodpoor A, Kolahi AA, Nejadghaderi SA, Sullman MJM, Mansournia MA, Ansarin K, Collins GS, Kaufman JS, Abdollahi M. 2022. Global burden of lower respiratory infections during the last three decades. Front Public Health 10:1028525. doi:10.3389/fpubh.2022.1028525 36699876 PMC9869262

[24] Rowe TA, Juthani-Mehta M. 2013. Urinary tract infection in older adults. Aging health 9. doi:10.2217/ahe.13.38 PMC3878051 24391677

[25] Leibovici-Weissman Y, Tau N, Yahav D. 2021. Bloodstream infections in the elderly: what is the real goal? Aging Clin Exp Res 33:1101–1112. doi:10.1007/s40520-019-01337-w 31486996

[26] Ursi MP, Durante Mangoni E, Rajani R, Hancock J, Chambers JB, Prendergast B. 2019. Infective endocarditis in the elderly: diagnostic and treatment options. Drugs Aging 36:115–124. doi:10.1007/s40266-018-0614-7 30488173

[27] Vella V, Derreumaux D, Aris E, Pellegrini M, Contorni M, Scherbakov M, Bagnoli F. 2024. The incidence of skin and soft tissue infections in the United States and associated healthcare utilization between 2010 and 2020. Open Forum Infect Dis 11:fae267. doi:10.1093/ofid/ofae267 PMC11146672 38835497

[28] Chong CP, Street PR. 2008. Pneumonia in the elderly: a review of the epidemiology, pathogenesis, microbiology, and clinical features. South Med J 101:1141–1145. doi:10.1097/SMJ.0b013e318181d5b5 19088525

[29] Simmons SR, Bhalla M, Herring SE, Tchalla EYI, Bou Ghanem EN. 2021. Older but not wiser: the age-driven changes in neutrophil responses during pulmonary infections. Infect Immun 89:e00653-20. doi:10.1128/IAI.00653-20 33495271 PMC8090953

[30] Mattison M. 2016. Hospital management of older adults, on Wolters Kluwer. Available from: https://www.uptodate.com/contents/hospital-management-of-older-adults. Retrieved 04 Oct 2016.

[31] High K. 2016. Evaluation of infection in the older adult. Available from: http://www.uptodate.com/contents/evaluation-of-infection-in-the-older-adult?source=see_link. Retrieved 04 Oct 2016.

[32] Mouton CP, Bazaldua OV, Pierce B, Espino DV. 2001. Common infections in older adults. Am Fam Physician 63:257–268. 11201692

[33] Talbird SE, La EM, Carrico J, Poston S, Poirrier JE, DeMartino JK, Hogea CS. 2021. Impact of population aging on the burden of vaccine-preventable diseases among older adults in the United States. Hum Vaccin Immunother 17:332–343. doi:10.1080/21645515.2020.1780847 32758069 PMC7899694

[34] Mackiewicz M, Nikonova EV, Zimmermann JE, Romer MA, Cater J, Galante RJ, Pack AI. 2006. Age-related changes in adenosine metabolic enzymes in sleep/wake regulatory areas of the brain. Neurobiol Aging 27:351–360. doi:10.1016/j.neurobiolaging.2005.01.015 16399217

[35] Willems L, Ashton KJ, Headrick JP. 2005. Adenosine-mediated cardioprotection in the aging myocardium. Cardiovasc Res 66:245–255. doi:10.1016/j.cardiores.2004.11.008 15820193

[36] Rabbani P, Ramkhelawon B, Cronstein BN. 2025. Adenosine metabolism and receptors in aging of the skin, musculoskeletal, immune and cardiovascular systems. Ageing Res Rev 106:102695. doi:10.1016/j.arr.2025.102695 39971100 PMC11960428

[37] Chang CP, Wu KC, Lin CY, Chern Y. 2021. Emerging roles of dysregulated adenosine homeostasis in brain disorders with a specific focus on neurodegenerative diseases. J Biomed Sci 28:70. doi:10.1186/s12929-021-00766-y 34635103 PMC8507231

[38] Marucci G, Buccioni M, Varlaro V, Volpini R, Amenta F. 2022. The possible role of the nucleoside adenosine in countering skin aging: a review. Biofactors 48:1027–1035. doi:10.1002/biof.1881 35979986 PMC9804842

[39] Czopik A, Yuan X, Evans SE, Eltzschig HK. 2021. Targeting the hypoxia-adenosine link for controlling excessive inflammation. Anesthesiology 135:15–17. doi:10.1097/ALN.0000000000003841 34046661 PMC8249341

[40] Boros D, Thompson J, Larson DF. 2016. Adenosine regulation of the immune response initiated by ischemia reperfusion injury. Perfusion 31:103–110. doi:10.1177/0267659115586579 25987550

[41] Eltzschig HK, Thompson LF, Karhausen J, Cotta RJ, Ibla JC, Robson SC, Colgan SP. 2004. Endogenous adenosine produced during hypoxia attenuates neutrophil accumulation: coordination by extracellular nucleotide metabolism. Blood 104:3986–3992. doi:10.1182/blood-2004-06-2066 15319286

[42] Antonioli L, Fornai M, Blandizzi C, Pacher P, Haskó G. 2019. Adenosine signaling and the immune system: when a lot could be too much. Immunol Lett 205:9–15. doi:10.1016/j.imlet.2018.04.006 29702147

[43] Godbout EJ, Madaline T, Casadevall A, Bearman G, Pirofski LA. 2020. The damage response framework and infection prevention: from concept to bedside. Infect Control Hosp Epidemiol 41:337–341. doi:10.1017/ice.2019.354 31915082

[44] Haskó G, Cronstein B. 2013. Regulation of inflammation by adenosine. Front Immunol 4:85. doi:10.3389/fimmu.2013.00085 23580000 PMC3619132

[45] Simmons SR, Herring SE, Tchalla EYI, Lenhard AP, Bhalla M, Bou Ghanem EN. 2024. Activating A1 adenosine receptor signaling boosts early pulmonary neutrophil recruitment in aged mice in response to Streptococcus pneumoniae infection. Immun Ageing 21:34. doi:10.1186/s12979-024-00442-3 38840213 PMC11151497

[46] Bhalla M, Hui Yeoh J, Lamneck C, Herring SE, Tchalla EYI, Heinzinger LR, Leong JM, Bou Ghanem EN. 2020. A1 adenosine receptor signaling reduces Streptococcus pneumoniae adherence to pulmonary epithelial cells by targeting expression of platelet‐activating factor receptor. Cell Microbiol 22:e13141. doi:10.1111/cmi.13141 31709673 PMC6980675

[47] Bhalla M, Simmons SR, Abamonte A, Herring SE, Roggensack SE, Bou Ghanem EN. 2020. Extracellular adenosine signaling reverses the age‐driven decline in the ability of neutrophils to kill Streptococcus pneumoniae. Aging Cell 19:e13218. doi:10.1111/acel.13218 32790148 PMC7576260

[48] Herring SE, Mao S, Bhalla M, Tchalla EYI, Kramer JM, Bou Ghanem EN. 2022. Mitochondrial ROS production by neutrophils is required for host antimicrobial function against Streptococcus pneumoniae and is controlled by A2B adenosine receptor signaling. PLoS Pathog 18:e1010700. doi:10.1371/journal.ppat.1010700 36374941 PMC9704767

[49] Barletta KE, Cagnina RE, Burdick MD, Linden J, Mehrad B. 2012. Adenosine A(2B) receptor deficiency promotes host defenses against gram-negative bacterial pneumonia. Am J Respir Crit Care Med 186:1044–1050. doi:10.1164/rccm.201204-0622OC 22997203 PMC3530209

[50] Barletta KE, Ley K, Mehrad B. 2012. Regulation of neutrophil function by adenosine. Arterioscler Thromb Vasc Biol 32:856–864. doi:10.1161/ATVBAHA.111.226845 22423037 PMC3353547

[51] Théâtre E, Frederix K, Guilmain W, Delierneux C, Lecut C, Bettendorff L, Bours V, Oury C. 2012. Overexpression of CD39 in mouse airways promotes bacteria-induced inflammation. J Immunol 189:1966–1974. doi:10.4049/jimmunol.1102600 22802412

[52] Nikolova M, Carriere M, Jenabian MA, Limou S, Younas M, Kok A, Hue S, Seddiki N, Hulin A, Delaneau O, Schuitemaker H, Herbeck JT, Mullins JI, Muhtarova M, Bensussan A, Zagury JF, Lelievre JD, Levy Y. 2011. CD39/adenosine pathway is involved in AIDS progression. PLoS Pathog 7:e1002110. doi:10.1371/journal.ppat.1002110 21750674 PMC3131268

[53] Jenabian M-A, Seddiki N, Yatim A, Carriere M, Hulin A, Younas M, Ghadimi E, Kök A, Routy J-P, Tremblay A, Sévigny J, Lelievre J-D, Levy Y. 2013. Regulatory T cells negatively affect IL-2 production of effector T cells through CD39/adenosine pathway in HIV infection. PLoS Pathog 9:e1003319. doi:10.1371/journal.ppat.1003319 23658513 PMC3635970

[54] Ravimohan S, Tamuhla N, Nfanyana K, Ni H, Steenhoff AP, Gross R, Weissman D, Bisson GP. 2017. Elevated pre-antiretroviral therapy CD39+CD8+ T cell frequency is associated with early mortality in advanced human immunodeficiency virus/tuberculosis co-infection. Clin Infect Dis 64:1453–1456. doi:10.1093/cid/cix155 28203772 PMC5411394

[55] Siwapornchai N, Lee JN, Tchalla EYI, Bhalla M, Yeoh JH, Roggensack SE, Leong JM, Bou Ghanem EN. 2020. Extracellular adenosine enhances the ability of PMNs to kill Streptococcus pneumoniae by inhibiting IL-10 production. J Leukoc Biol 108:867–882. doi:10.1002/JLB.4MA0120-115RR 32017200 PMC8314384

[56] Bou Ghanem EN, Clark S, Roggensack SE, McIver SR, Alcaide P, Haydon PG, Leong JM. 2015. Extracellular adenosine protects against Streptococcus pneumoniae lung infection by regulating pulmonary neutrophil recruitment. PLoS Pathog 11:e1005126. doi:10.1371/journal.ppat.1005126 26313746 PMC4552087

[57] Petit-Jentreau L, Jouvion G, Charles P, Majlessi L, Gicquel B, Tailleux L. 2015. Ecto-5’-nucleotidase (CD73) deficiency in Mycobacterium tuberculosis-infected mice enhances neutrophil recruitment. Infect Immun 83:3666–3674. doi:10.1128/IAI.00418-15 26150535 PMC4534646

[58] Alam MS, Kurtz CC, Rowlett RM, Reuter BK, Wiznerowicz E, Das S, Linden J, Crowe SE, Ernst PB. 2009. CD73 is expressed by human regulatory T helper cells and suppresses proinflammatory cytokine production and Helicobacter felis –induced gastritis in mice. J Infect Dis 199:494–504. doi:10.1086/596205 19281303 PMC3047419

[59] Alam MS, Kuo JL, Ernst PB, Derr-Castillo V, Pereira M, Gaines D, Costales M, Bigley E, Williams K. 2014. Ecto-5’-nucleotidase (CD73) regulates host inflammatory responses and exacerbates murine salmonellosis. Sci Rep 4:4486. doi:10.1038/srep04486 24670982 PMC3967249

[60] Francois V, Shehade H, Acolty V, Preyat N, Delrée P, Moser M, Oldenhove G. 2015. Intestinal immunopathology is associated with decreased CD73-generated adenosine during lethal infection. Mucosal Immunol 8:773–784. doi:10.1038/mi.2014.108 25389034

[61] Gallos G, Ruyle TD, Emala CW, Lee HT. 2005. A1 adenosine receptor knockout mice exhibit increased mortality, renal dysfunction, and hepatic injury in murine septic peritonitis. Am J Physiol Renal Physiol 289:F369–76. doi:10.1152/ajprenal.00470.2004 15784841

[62] Wilson CN, Vance CO, Lechner MG, Matuschak GM, Lechner AJ. 2014. Adenosine A1 receptor antagonist, L-97-1, improves survival and protects the kidney in a rat model of cecal ligation and puncture induced sepsis. Eur J Pharmacol 740:346–352. doi:10.1016/j.ejphar.2014.07.012 25041842 PMC4147868

[63] Aeffner F, Woods PS, Davis IC. 2014. Activation of A1-adenosine receptors promotes leukocyte recruitment to the lung and attenuates acute lung injury in mice infected with influenza A/WSN/33 (H1N1) virus. J Virol 88:10214–10227. 24965449 10.1128/JVI.01068-14 PMC4136329

[64] Costa DVS, Shin JH, Goldbeck SM, Bolick DT, Mesquita FS, Loureiro AV, Rodrigues-Jesus MJ, Brito GAC, Warren CA. 2022. Adenosine receptors differentially mediate enteric glial cell death induced by Clostridioides difficile toxins A and B. Front Immunol 13:956326. doi:10.3389/fimmu.2022.956326 36726986 PMC9885079

[65] Li Y, Figler RA, Kolling G, Bracken TC, Rieger J, Stevenson RW, Linden J, Guerrant RL, Warren CA. 2012. Adenosine A2A receptor activation reduces recurrence and mortality from Clostridium difficile infection in mice following vancomycin treatment. BMC Infect Dis 12:342. doi:10.1186/1471-2334-12-342 23217055 PMC3523970

[66] Foschetti DA, Braga-Neto MB, Bolick D, Moore J, Alves LA, Martins CS, Bomfin LE, Santos A, Leitão R, Brito G, Warren CA. 2020. Clostridium difficile toxins or infection induce upregulation of adenosine receptors and IL-6 with early pro-inflammatory and late anti-inflammatory pattern. Braz J Med Biol Res 53:e9877. doi:10.1590/1414-431x20209877 32725081 PMC7405017

[67] Warren CA, Calabrese GM, Li Y, Pawlowski SW, Figler RA, Rieger J, Ernst PB, Linden J, Guerrant RL. 2012. Effects of adenosine A(2)A receptor activation and alanyl-glutamine in Clostridium difficile toxin-induced ileitis in rabbits and cecitis in mice. BMC Infect Dis 12:13. doi:10.1186/1471-2334-12-13 22264229 PMC3323464

[68] Sullivan GW, Fang G, Linden J, Scheld WM. 2004. A2A adenosine receptor activation improves survival in mouse models of endotoxemia and sepsis. J Infect Dis 189:1897–1904. doi:10.1086/386311 15122527

[69] Wu XN, Dong K, Liu Y, Yang L, Zhang J, Yang M, Gao ZW. 2025. Adenosine A2A receptor activation alleviated disease of mice with systemic Candida albicans infection by regulating macrophage function. J Inflamm Res 18:3283–3294. doi:10.2147/JIR.S501546 40065909 PMC11892491

[70] Ko MK, Shao H, Kaplan HJ, Sun D. 2021. Timing effect of adenosine-directed immunomodulation on mouse experimental autoimmune uveitis. J Immunol 207:153–161. doi:10.4049/jimmunol.2100182 34127521 PMC8669050

[71] Monticone G, Huang Z, Hewins P, Cook T, Mirzalieva O, King B, Larter K, Miller-Ensminger T, Sanchez-Pino MD, Foster TP, Nichols OV, Ramsay AJ, Majumder S, Wyczechowska D, Tauzier D, Gravois E, Crabtree JS, Garai J, Li L, Zabaleta J, Barbier MT, Del Valle L, Jurado KA, Miele L. 2024. Novel immunomodulatory properties of adenosine analogs promote their antiviral activity against SARS-CoV-2. EMBO Rep 25:3547–3573. doi:10.1038/s44319-024-00189-4 39009832 PMC11315900

[72] Vozza EG, Daly CM, O’Rourke SA, Fitzgerald HK, Dunne A, McLoughlin RM. 2024. Staphylococcus aureus suppresses the pentose phosphate pathway in human neutrophils via the adenosine receptor A2aR to enhance intracellular survival. mBio 15:e02571-23. doi:10.1128/mbio.02571-23 38108639 PMC10790693

[73] Liu P, Pian Y, Li X, Liu R, Xie W, Zhang C, Zheng Y, Jiang Y, Yuan Y. 2014. Streptococcus suis adenosine synthase functions as an effector in evasion of pmn-mediated innate immunity. J Infect Dis 210:35–45. doi:10.1093/infdis/jiu050 24446521

[74] Spooner R, DeGuzman J, Lee KL, Yilmaz Ö. 2014. Danger signal adenosine via adenosine 2a receptor stimulates growth of porphyromonas gingivalis in primary gingival epithelial cells. Mol Oral Microbiol 29:67–78. doi:10.1111/omi.12045 24517244 PMC3960722

[75] Warren CA, Li Y, Calabrese GM, Freire RS, Zaja-Milatovic S, van Opstal E, Figler RA, Linden J, Guerrant RL. 2012. Contribution of adenosine A(2B) receptors in Clostridium difficile intoxication and infection. Infect Immun 80:4463–4473. doi:10.1128/IAI.00782-12 23045479 PMC3497433

[76] Tang W, Guan M, Li Z, Pan W, Wang Z. 2023. A2BR facilitates the pathogenesis of H. pylori-associated GU by inducing oxidative stress through p38 MAPK phosphorylation. Heliyon 9:e21004. doi:10.1016/j.heliyon.2023.e21004 38027590 PMC10660004

[77] Belikoff BG, Hatfield S, Georgiev P, Ohta A, Lukashev D, Buras JA, Remick DG, Sitkovsky M. 2011. A2B adenosine receptor blockade enhances macrophage-mediated bacterial phagocytosis and improves polymicrobial sepsis survival in mice. J Immunol 186:2444–2453. doi:10.4049/jimmunol.1001567 21242513 PMC3708265

[78] Csóka B, Németh ZH, Rosenberger P, Eltzschig HK, Spolarics Z, Pacher P, Selmeczy Z, Koscsó B, Himer L, Vizi ES, Blackburn MR, Deitch EA, Haskó G. 2010. A2B adenosine receptors protect against sepsis-induced mortality by dampening excessive inflammation. J Immunol 185:542–550. doi:10.4049/jimmunol.0901295 20505145 PMC2938184

[79] Figueiredo AB, de Oliveira E Castro RA, Nogueira-Paiva NC, Moreira F, Gonçalves FQ, Soares RP, Castro-Borges W, Silva GG, Cunha RA, Gonçalves T, Afonso LCC. 2021. Clustering of adenosine A(2B) receptors with ectonucleotidases in caveolin-rich lipid rafts underlies immunomodulation by Leishmania amazonensis. FASEB J 35:e21509. doi:10.1096/fj.202002396RR 33813781

[80] Figueiredo AB, Serafim TD, Marques-da-Silva EA, Meyer-Fernandes JR, Afonso LC. 2012. Leishmania amazonensis impairs DC function by inhibiting CD40 expression via A2B adenosine receptor activation. Eur J Immunol 42:1203–1215. doi:10.1002/eji.201141926 22311598

[81] Figueiredo AB, Souza-Testasicca MC, Mineo TWP, Afonso LCC. 2017. Leishmania amazonensis-induced cAMP triggered by adenosine A(2B) receptor is important to inhibit dendritic cell activation and evade immune response in infected mice. Front Immunol 8:849. doi:10.3389/fimmu.2017.00849 28791011 PMC5524897

[82] Patel N, Wu W, Mishra PK, Chen F, Millman A, Csóka B, Koscsó B, Eltzschig HK, Haskó G, Gause WC. 2014. A2B adenosine receptor induces protective antihelminth type 2 immune responses. Cell Host Microbe 15:339–350. doi:10.1016/j.chom.2014.02.001 24629340

[83] Inoue Y, Chen Y, Hirsh MI, Yip L, Junger WG. 2008. A3 and P2Y2 receptors control the recruitment of neutrophils to the lungs in a mouse model of sepsis. Shock 30:173–177. doi:10.1097/shk.0b013e318160dad4 18091570 PMC4212521

[84] Xia C, Yin S, To KKW, Fu L. 2023. CD39/CD73/A2AR pathway and cancer immunotherapy. Mol Cancer 22:44. doi:10.1186/s12943-023-01733-x 36859386 PMC9979453

[85] Antonioli L, Pacher P, Vizi ES, Haskó G. 2013. CD39 and CD73 in immunity and inflammation. Trends Mol Med 19:355–367. doi:10.1016/j.molmed.2013.03.005 23601906 PMC3674206

[86] Borsellino G, Kleinewietfeld M, Di Mitri D, Sternjak A, Diamantini A, Giometto R, Höpner S, Centonze D, Bernardi G, Dell’Acqua ML, Rossini PM, Battistini L, Rötzschke O, Falk K. 2007. Expression of ectonucleotidase CD39 by Foxp3+ Treg cells: hydrolysis of extracellular ATP and immune suppression. Blood 110:1225–1232. doi:10.1182/blood-2006-12-064527 17449799

[87] Ohta A, Sitkovsky M. 2014. Extracellular adenosine-mediated modulation of regulatory T cells. Front Immunol 5:304. doi:10.3389/fimmu.2014.00304 25071765 PMC4091046

[88] Dorneles GP, Teixeira PC, Silva IM, Schipper LL, Santana Filho PC, Rodrigues Junior LC, Bonorino C, Peres A, Fonseca SG, Monteiro MC, Boeck CR, Eller S, Oliveira TF, Wendland EM, Romao PRT. 2022. Alterations in CD39/CD73 axis of T cells associated with COVID-19 severity. J Cell Physiol 237:3394–3407. doi:10.1002/jcp.30805 35754396 PMC9349448

[89] Elsaghir A, El-Sabaa EMW, Zahran AM, Mandour SA, Salama EH, Aboulfotuh S, El-Morshedy RM, Tocci S, Mandour AM, Ali WE, Abdel-Wahid L, Sayed IM, El-Mokhtar MA. 2023. Elevated CD39+T-regulatory cells and reduced levels of adenosine indicate a role for tolerogenic signals in the progression from moderate to severe COVID-19. Int J Mol Sci 24:17614. doi:10.3390/ijms242417614 38139439 PMC10744088

[90] Amaral EP, Machado de Salles É, Barbosa Bomfim CC, Salgado RM, Almeida FM, de Souza PC, Alvarez JM, Hirata MH, Lasunskaia EB, D’Império-Lima MR. 2019. Inhibiting adenosine receptor signaling promotes accumulation of effector CD4+ T cells in the lung parenchyma during severe tuberculosis. J Infect Dis 219:964–974. doi:10.1093/infdis/jiy586 30307561

[91] Mahamed DA, Mills JH, Egan CE, Denkers EY, Bynoe MS. 2012. CD73-generated adenosine facilitates Toxoplasma gondii differentiation to long-lived tissue cysts in the central nervous system. Proc Natl Acad Sci USA 109:16312–16317. doi:10.1073/pnas.1205589109 22988118 PMC3479617

[92] Aeffner F, Woods PS, Davis IC. 2015. Ecto-5’-nucleotidase CD73 modulates the innate immune response to influenza infection but is not required for development of influenza-induced acute lung injury. Am J Physiol Lung Cell Mol Physiol 309:L1313–L1322. doi:10.1152/ajplung.00130.2015 26432867 PMC4669338

[93] Wolk KE, Lazarowski ER, Traylor ZP, Yu ENZ, Jewell NA, Durbin RK, Durbin JE, Davis IC. 2008. Influenza A virus inhibits alveolar fluid clearance in BALB/c mice. Am J Respir Crit Care Med 178:969–976. doi:10.1164/rccm.200803-455OC 18689466 PMC2577730

[94] Wilson CN, Vance CO, Doyle TM, Brink DS, Matuschak GM, Lechner AJ. 2012. A novel post-exposure medical countermeasure L-97-1 improves survival and acute lung injury following intratracheal infection with Yersinia pestis. Innate Immun 18:373–389. doi:10.1177/1753425911411595 21862597 PMC3362682

[95] Alam MS, Kurtz CC, Wilson JM, Burnette BR, Wiznerowicz EB, Ross WG, Rieger JM, Figler RA, Linden J, Crowe SE, Ernst PB. 2009. A2A adenosine receptor (AR) activation inhibits pro-inflammatory cytokine production by human CD4+ helper T cells and regulates Helicobacter-induced gastritis and bacterial persistence. Mucosal Immunol 2:232–242. doi:10.1038/mi.2009.4 19262506 PMC3036970

[96] Rogachev B, Ziv NY, Mazar J, Nakav S, Chaimovitz C, Zlotnik M, Douvdevani A. 2006. Adenosine is upregulated during peritonitis and is involved in downregulation of inflammation. Kidney Int 70:675–681. doi:10.1038/sj.ki.5001609 16788688

[97] Li X, Liang D, Shao H, Born WK, Kaplan HJ, Sun D. 2019. Adenosine receptor activation in the Th17 autoimmune responses of experimental autoimmune uveitis. Cell Immunol 339:24–28. doi:10.1016/j.cellimm.2018.09.004 30249343

[98] Tokano M, Matsushita S, Takagi R, Yamamoto T, Kawano M. 2022. Extracellular adenosine induces hypersecretion of IL-17A by T-helper 17 cells through the adenosine A2a receptor. Brain Behav Immun Health 26:100544. doi:10.1016/j.bbih.2022.100544 36467126 PMC9712818

[99] Vijayamahantesh S Amit A, Kumar S, Dikhit MR, Jha PK, Singh AK, Sinha KK, Pandey K, Das VNR, Das P, Bimal S. 2016. Up regulation of A2B adenosine receptor on monocytes are crucially required for immune pathogenicity in Indian patients exposed to Leishmania donovani. Cytokine 79:38–44. doi:10.1016/j.cyto.2015.12.016 26748211

[100] Caporarello N, Olivieri M, Cristaldi M, Scalia M, Toscano MA, Genovese C, Addamo A, Salmeri M, Lupo G, Anfuso CD. 2018. Blood–brain barrier in a Haemophilus influenzae type a in vitro infection: role of adenosine receptors A2A and A2B. Mol Neurobiol 55:5321–5336. doi:10.1007/s12035-017-0769-y 28921456

[101] Németh ZH, Csóka B, Wilmanski J, Xu D, Lu Q, Ledent C, Deitch EA, Pacher P, Spolarics Z, Haskó G. 2006. Adenosine A2A receptor inactivation increases survival in polymicrobial sepsis. J Immunol 176:5616–5626. doi:10.4049/jimmunol.176.9.5616 16622031 PMC2268092

[102] Eckle T, Kewley EM, Brodsky KS, Tak E, Bonney S, Gobel M, Anderson D, Glover LE, Riegel AK, Colgan SP, Eltzschig HK. 2014. Identification of hypoxia-inducible factor HIF-1A as transcriptional regulator of the A2B adenosine receptor during acute lung injury. J Immunol 192:1249–1256. doi:10.4049/jimmunol.1100593 24391213 PMC3946986

[103] Kong T, Westerman KA, Faigle M, Eltzschig HK, Colgan SP. 2006. HIF‐dependent induction of adenosine A2B receptor in hypoxia. FASEB J 20:2242–2250. doi:10.1096/fj.06-6419com 17077301

[104] dos Santos PMF, Díaz Acosta CC, Rosa TLSA, Ishiba MH, Dias AA, Pereira AMR, Gutierres LD, Pereira MP, da Silva Rocha M, Rosa PS, Bertoluci DFF, Meyer-Fernandes JR, da Mota Ramalho Costa F, Marques MAM, Belisle JT, Pinheiro RO, Rodrigues LS, Pessolani MCV, Berrêdo-Pinho M. 2024. Adenosine A2A receptor as a potential regulator of Mycobacterium leprae survival mechanisms: new insights into leprosy neural damage. Front Pharmacol 15:1399363. doi:10.3389/fphar.2024.1399363 39005937 PMC11239521

[105] Salvatore CA, Jacobson MA, Taylor HE, Linden J, Johnson RG. 1993. Molecular cloning and characterization of the human A3 adenosine receptor. Proc Natl Acad Sci USA 90:10365–10369. doi:10.1073/pnas.90.21.10365 8234299 PMC47775

[106] Chen Y, Corriden R, Inoue Y, Yip L, Hashiguchi N, Zinkernagel A, Nizet V, Insel PA, Junger WG. 2006. ATP release guides neutrophil chemotaxis via P2Y2 and A3 receptors. Science 314:1792–1795. doi:10.1126/science.1132559 17170310

[107] Wagner R, Ngamsri KC, Stark S, Vollmer I, Reutershan J. 2010. Adenosine receptor A3 is a critical mediator in LPS-induced pulmonary inflammation. Am J Physiol Lung Cell Mol Physiol 299:L502–12. doi:10.1152/ajplung.00083.2010 20639349

[108] Jordan JE, Thourani VH, Auchampach JA, Robinson JA, Wang NP, Vinten-Johansen J. 1999. A(3) adenosine receptor activation attenuates neutrophil function and neutrophil-mediated reperfusion injury. Am J Physiol 277:H1895–905. doi:10.1152/ajpheart.1999.277.5.H1895 10564145

[109] Riedmaier P, Sansom FM, Sofian T, Beddoe T, Schuelein R, Newton HJ, Hartland EL. 2014. Multiple ecto-nucleoside triphosphate diphosphohydrolases facilitate intracellular replication of Legionella pneumophila. Biochem J 462:279–289. doi:10.1042/BJ20130923 24957128

[110] Sansom FM, Newton HJ, Crikis S, Cianciotto NP, Cowan PJ, d’Apice AJF, Hartland EL. 2007. A bacterial ecto-triphosphate diphosphohydrolase similar to human CD39 is essential for intracellular multiplication of Legionella pneumophila. Cell Microbiol 9:1922–1935. doi:10.1111/j.1462-5822.2007.00924.x 17388784

[111] Thammavongsa V, Kern JW, Missiakas DM, Schneewind O. 2009. Staphylococcus aureus synthesizes adenosine to escape host immune responses. J Exp Med 206:2417–2427. doi:10.1084/jem.20090097 19808256 PMC2768845

[112] Zhao Z, Shang X, Chen Y, Zheng Y, Huang W, Jiang H, Lv Q, Kong D, Jiang Y, Liu P. 2020. Bacteria elevate extracellular adenosine to exploit host signaling for blood-brain barrier disruption. Virulence 11:980–994. doi:10.1080/21505594.2020.1797352 32772676 PMC7549952

[113] Firon A, Dinis M, Raynal B, Poyart C, Trieu-Cuot P, Kaminski PA. 2014. Extracellular nucleotide catabolism by the group B Streptococcus ectonucleotidase NudP increases bacterial survival in blood. J Biol Chem 289:5479–5489. doi:10.1074/jbc.M113.545632 24429288 PMC3937624

[114] Fan J, Zhang Y, Chuang-Smith ON, Frank KL, Guenther BD, Kern M, Schlievert PM, Herzberg MC. 2012. Ecto-5′-nucleotidase: a candidate virulence factor in Streptococcus sanguinis experimental endocarditis. PLoS One 7:e38059. doi:10.1371/journal.pone.0038059 22685551 PMC3369921

[115] López-Otín C, Blasco MA, Partridge L, Serrano M, Kroemer G. 2023. Hallmarks of aging: an expanding universe. Cell 186:243–278. doi:10.1016/j.cell.2022.11.001 36599349

[116] Stupka JE, Mortensen EM, Anzueto A, Restrepo MI. 2009. Community-acquired pneumonia in elderly patients. Aging health 5:763–774. doi:10.2217/ahe.09.74 20694055 PMC2917114

[117] Ruiz LA, España PP, Gómez A, Bilbao A, Jaca C, Arámburu A, Capelastegui A, Restrepo MI, Zalacain R. 2017. Age-related differences in management and outcomes in hospitalized healthy and well-functioning bacteremic pneumococcal pneumonia patients: a cohort study. BMC Geriatr 17:130. doi:10.1186/s12877-017-0518-0 28633626 PMC5477680

[118] Krone CL, van de Groep K, Trzciński K, Sanders EAM, Bogaert D. 2014. Immunosenescence and pneumococcal disease: an imbalance in host-pathogen interactions. Lancet Respir Med 2:141–153. doi:10.1016/S2213-2600(13)70165-6 24503269

[119] Hodes RJ, Sierra F, Austad SN, Epel E, Neigh GN, Erlandson KM, Schafer MJ, LeBrasseur NK, Wiley C, Campisi J, Sehl ME, Scalia R, Eguchi S, Kasinath BS, Halter JB, Cohen HJ, Demark-Wahnefried W, Ahles TA, Barzilai N, Hurria A, Hunt PW. 2016. Disease drivers of aging. Ann N Y Acad Sci 1386:45–68. doi:10.1111/nyas.13299 27943360 PMC5373660

[120] Sendama W. 2020. The effect of ageing on the resolution of inflammation. Ageing Res Rev 57:101000. doi:10.1016/j.arr.2019.101000 31862417 PMC6961112

[121] Ledderose C, Valsami EA, Elevado M, Liu Q, Giva B, Curatolo J, Delfin J, Abutabikh R, Junger WG. 2024. Impaired ATP hydrolysis in blood plasma contributes to age-related neutrophil dysfunction. Immun Ageing 21:45. doi:10.1186/s12979-024-00441-4 38961477 PMC11221114

[122] Crooke A, Martínez-Henández J, Martínez-López J, Cruz-Jentoft A, Huete-Toral F, Pintor J. 2017. Low expression of CD39 and CD73 genes in centenarians compared with octogenarians. Immun Ageing 14:11. doi:10.1186/s12979-017-0094-3 28529533 PMC5437401

[123] Hesdorffer CS, Malchinkhuu E, Biragyn A, Mabrouk OS, Kennedy RT, Madara K, Taub DD, Longo DL, Schwartz JB, Ferrucci L, Goetzl EJ. 2012. Distinctive immunoregulatory effects of adenosine on T cells of older humans. FASEB J 26:1301–1310. doi:10.1096/fj.11-197046 22121051 PMC3289505

[124] Fang F, Cao W, Zhu W, Lam N, Li L, Gaddam S, Wang Y, Kim C, Lambert S, Zhang H, Hu B, Farber DL, Weyand CM, Goronzy JJ. 2021. The cell-surface 5’-nucleotidase CD73 defines a functional T memory cell subset that declines with age. Cell Rep 37:109981. doi:10.1016/j.celrep.2021.109981 34758299 PMC8612175

[125] Jeske SS, Schuler PJ, Doescher J, Theodoraki MN, Laban S, Brunner C, Hoffmann TK, Wigand MC. 2020. Age-related changes in T lymphocytes of patients with head and neck squamous cell carcinoma. Immun Ageing 17:3. doi:10.1186/s12979-020-0174-7 32082401 PMC7017629

[126] Fang F, Yu M, Cavanagh MM, Hutter Saunders J, Qi Q, Ye Z, Le Saux S, Sultan W, Turgano E, Dekker CL, Tian L, Weyand CM, Goronzy JJ. 2016. Expression of CD39 on activated T cells impairs their survival in older individuals. Cell Rep 14:1218–1231. doi:10.1016/j.celrep.2016.01.002 26832412 PMC4851554

[127] Alam MS, Cavanaugh C, Pereira M, Babu U, Williams K. 2020. Susceptibility of aging mice to listeriosis: role of anti-inflammatory responses with enhanced treg-cell expression of CD39/CD73 and Th-17 cells. Int J Med Microbiol 310:151397. doi:10.1016/j.ijmm.2020.151397 31974050

[128] Rodrigues L, Miranda IM, Andrade GM, Mota M, Cortes L, Rodrigues AG, Cunha RA, Gonçalves T. 2016. Blunted dynamics of adenosine A2A receptors is associated with increased susceptibility to Candida albicans infection in the elderly. Oncotarget 7:62862–62872. doi:10.18632/oncotarget.11760 27590517 PMC5325332

[129] Eltzschig HK, Macmanus CF, Colgan SP. 2008. Neutrophils as sources of extracellular nucleotides: functional consequences at the vascular interface. Trends Cardiovasc Med 18:103–107. doi:10.1016/j.tcm.2008.01.006 18436149 PMC2711033

[130] van Waeg G, Van den Berghe G. 1991. Purine catabolism in polymorphonuclear neutrophils. Phorbol myristate acetate-induced accumulation of adenosine owing to inactivation of extracellularly released adenosine deaminase. J Clin Invest 87:305–312. doi:10.1172/JCI114987 1898656 PMC295052

[131] Palatella M, Guillaume SM, Linterman MA, Huehn J. 2022. The dark side of Tregs during aging. Front Immunol 13:940705. doi:10.3389/fimmu.2022.940705 36016952 PMC9398463

[132] Parish ST, Kim S, Sekhon RK, Wu JE, Kawakatsu Y, Effros RB. 2010. Adenosine deaminase modulation of telomerase activity and replicative senescence in human CD8 T lymphocytes. J Immunol 184:2847–2854. doi:10.4049/jimmunol.0903647 20147632 PMC3772624

[133] Crosti F, Ciboddo GF, Barbieri MC, Inversi F, Pavoni D, Quarenghi S, Navone P, Vezzoni P, Rugarli C. 1987. Evidence for adenosine adenosinedeaminase lymphocyte system impairment in ageing. Boll Ist Sieroter Milan 66:282–288. 3442618

[134] Grant LR, Meche A, McGrath L, Miles A, Alfred T, Yan Q, Chilson E. 2023. Risk of pneumococcal disease in US adults by age and risk profile. Open Forum Infect Dis 10:fad192. doi:10.1093/ofid/ofad192 PMC10167987 37180598

[135] Bartleson JM, Radenkovic D, Covarrubias AJ, Furman D, Winer DA, Verdin E. 2021 SARS-CoV-2, COVID-19 and the aging immune system. Nat Aging 1:769–782. doi:10.1038/s43587-021-00114-7 34746804 PMC8570568

[136] Wambogo EA, Vaudin AM, Moshfegh AJ, Spungen JH, Doren JMV, Sahyoun NR. 2020. Toward a better understanding of listeriosis risk among older adults in the united states: characterizing dietary patterns and the sociodemographic and economic attributes of consumers with these patterns. J Food Prot 83:1208–1217. doi:10.4315/JFP-19-617 32221521

[137] Dekkers BGJ, Veringa A, Marriott DJE, Boonstra JM, van der Elst KCM, Doukas FF, McLachlan AJ, Alffenaar J-WC. 2018. Invasive candidiasis in the elderly: considerations for drug therapy. Drugs Aging 35:781–789. doi:10.1007/s40266-018-0576-9 30047069 PMC6105183

[138] Laflamme C, Mailhot GB, Pouliot M. 2017. Age-related decline of the acute local inflammation response: a mitigating role for the adenosine A(2A) receptor. Aging 9:2083–2097. doi:10.18632/aging.101303 29064819 PMC5680557

[139] Bou Ghanem EN, Clark S, Du X, Wu D, Camilli A, Leong JM, Meydani SN. 2015. The α-tocopherol form of vitamin E reverses age-associated susceptibility to Streptococcus pneumoniae lung infection by modulating pulmonary neutrophil recruitment. J Immunol 194:1090–1099. doi:10.4049/jimmunol.1402401 25512603 PMC4834212

[140] Simmons SR, Lenhard AP, Battaglia MC, Bou Ghanem EN. 2025. Adenosine 2B receptor signaling impairs vaccine-mediated protection against pneumococcal infection in young hosts by blunting neutrophil killing of antibody-opsonized bacteria. Vaccines 13:414. doi:10.3390/vaccines13040414 40333334 PMC12031446

[141] Ledderose C, Valsami EA, Newhams M, Elevado MJ, Novak T, Randolph AG, Junger WG. 2023. ATP breakdown in plasma of children limits the antimicrobial effectiveness of their neutrophils. Purinergic Signal 19:651–662. doi:10.1007/s11302-022-09915-w 36596963 PMC10754799

[142] Kaufmann I, Hoelzl A, Schliephake F, Hummel T, Chouker A, Łysenko L, Peter K, Thiel M. 2007. Effects of adenosine on functions of polymorphonuclear leukocytes from patients with septic shock. Shock 27:25–31. doi:10.1097/01.shk.0000238066.00074.90 17172976

[143] Ramakers BP, Wever KE, Kox M, van den Broek PH, Mbuyi F, Rongen G, Masereeuw R, van der Hoeven JG, Smits P, Riksen NP, Pickkers P. 2012. How systemic inflammation modulates adenosine metabolism and adenosine receptor expression in humans in vivo. Crit Care Med 40:2609–2616. doi:10.1097/CCM.0b013e318259205b 22732294

[144] Sun CX, Wall NR, Angelov N, Ririe C, Chen JW, Boskovic DS, Henkin JM. 2011. Changes in mRNA expression of adenosine receptors in human chronic periodontitis. Chin J Dent Res 14:113–120. 22319752

[145] Kim SH, Kim YK, Park HW, Kim SH, Kim SH, Ye YM, Min KU, Park HS. 2009. Adenosine deaminase and adenosine receptor polymorphisms in aspirin-intolerant asthma. Respir Med 103:356–363. doi:10.1016/j.rmed.2008.10.008 19019667

[146] Hider SL, Thomson W, Mack LF, Armstrong DJ, Shadforth M, Bruce IN. 2008. Polymorphisms within the adenosine receptor 2a gene are associated with adverse events in RA patients treated with MTX. Rheumatology 47:1156–1159. doi:10.1093/rheumatology/ken182 18539621 PMC2468887

[147] Stampanoni Bassi M, Buttari F, Simonelli I, Gilio L, Furlan R, Finardi A, Marfia GA, Visconti A, Paolillo A, Storto M, Gambardella S, Ferese R, Salvetti M, Uccelli A, Matarese G, Centonze D, De Vito F. 2020. A single nucleotide ADA genetic variant is associated to central inflammation and clinical presentation in MS: implications for cladribine treatment. Genes 11:1152. doi:10.3390/genes11101152 33007809 PMC7601054

[148] Peng HX, Zhang LL, Jiang D, Jian N, Zhang TM, Luo JG, Yin HY. 2024. CD73 polymorphisms are associated with schizophrenia. Purinergic Signal. doi:10.1007/s11302-024-10004-3 PMC12454215 38758511

[149] Friedman DJ, Künzli BM, A-Rahim YI, Sevigny J, Berberat PO, Enjyoji K, Csizmadia E, Friess H, Robson SC. 2009. From the Cover: CD39 deletion exacerbates experimental murine colitis and human polymorphisms increase susceptibility to inflammatory bowel disease. Proc Natl Acad Sci USA 106:16788–16793. doi:10.1073/pnas.0902869106 19805374 PMC2757811

[150] Lindo J, Nogueira C, Soares R, Cunha N, Almeida MR, Rodrigues L, Coelho P, Rodrigues F, Cunha RA, Gonçalves T. 2024. Genetic polymorphisms of P2RX7 but not of ADORA2A are associated with the severity of SARS-CoV-2 infection. Int J Mol Sci 25:6135. doi:10.3390/ijms25116135 38892324 PMC11173306

[151] Gupta H, Jain A, Saadi AV, Vasudevan TG, Hande MH, D’Souza SC, Ghosh SK, Umakanth S, Satyamoorthy K. 2015. Categorical complexities of Plasmodium falciparum malaria in individuals is associated with genetic variations in ADORA2A and GRK5 genes. Infect Genet Evol 34:188–199. doi:10.1016/j.meegid.2015.06.010 26066465

[152] Cannata A, De Luca C, Korkina LG, Ferlazzo N, Ientile R, Currò M, Andolina G, Caccamo D. 2020. The SNP rs2298383 reduces ADORA2A gene transcription and positively associates with cytokine production by peripheral blood mononuclear cells in patients with multiple chemical sensitivity. Int J Mol Sci 21:1858. doi:10.3390/ijms21051858 32182774 PMC7084623

[153] Zhong F, Dai T, Sheng Y. 2018. Correlation study between ADA and IFN-γ gene polymorphisms and the risk of developing tuberculous pericarditis. Gene 676:214–218. doi:10.1016/j.gene.2018.07.032 30017738

[154] Ramakers BP, Giamarellos-Bourboulis EJ, Tasioudis C, Coenen MJH, Kox M, Vermeulen SH, Groothuismink JM, van der Hoeven JG, Routsi C, Savva A, Prekates A, Diamantea F, Sinapidis D, Smits P, Toutouzas K, Riksen NP, Pickkers P. 2015. Effects of the 34C>T variant of the AMPD1 gene on immune function, multi-organ dysfunction, and mortality in sepsis patients. Shock 44:542–547. doi:10.1097/SHK.0000000000000456 26529652

[155] Chen JF, Eltzschig HK, Fredholm BB. 2013. Adenosine receptors as drug targets--what are the challenges? Nat Rev Drug Discov 12:265–286. doi:10.1038/nrd3955 23535933 PMC3930074

[156] Vincenzi F, Pasquini S, Contri C, Cappello M, Nigro M, Travagli A, Merighi S, Gessi S, Borea PA, Varani K. 2023. Pharmacology of adenosine receptors: recent advancements. Biomolecules 13:1387. doi:10.3390/biom13091387 37759787 PMC10527030

[157] Al Jaroudi W, Iskandrian AE. 2009. Regadenoson: a new myocardial stress agent. J Am Coll Cardiol 54:1123–1130. doi:10.1016/j.jacc.2009.04.089 19761931

[158] Lieu HD, Shryock JC, von Mering GO, Gordi T, Blackburn B, Olmsted AW, Belardinelli L, Kerensky RA. 2007. Regadenoson, a selective A2A adenosine receptor agonist, causes dose-dependent increases in coronary blood flow velocity in humans. J Nucl Cardiol 14:514–520. doi:10.1016/j.nuclcard.2007.02.016 17679059

[159] Berger AA, Winnick A, Welschmeyer A, Kaneb A, Berardino K, Cornett EM, Kaye AD, Viswanath O, Urits I. 2020. Istradefylline to treat patients with parkinson’s disease experiencing “Off” episodes: a comprehensive review. Neurol Int 12:109–129. doi:10.3390/neurolint12030017 33302331 PMC7768423

[160] Rabin J, Zhao Y, Mostafa E, Al-Suqi M, Fleischmann E, Conaway MR, Mann BJ, Chhabra P, Brayman KL, Krupnick A, Linden J, Lau CL. 2023. Regadenoson for the treatment of COVID-19: a five case clinical series and mouse studies. PLoS One 18:e0288920. doi:10.1371/journal.pone.0288920 37566593 PMC10420352

[161] Mathur P, Kottilil S, Wilson E. 2018. Use of ribavirin for hepatitis C treatment in the modern direct-acting antiviral era. J Clin Transl Hepatol 6:431–437. doi:10.14218/JCTH.2018.00007 30637222 PMC6328726

[162] Jacobson KA, Tosh DK, Jain S, Gao ZG. 2019. Historical and current adenosine receptor agonists in preclinical and clinical development. Front Cell Neurosci 13:124. doi:10.3389/fncel.2019.00124 30983976 PMC6447611

[163] Peleli M, Fredholm BB, Sobrevia L, Carlström M. 2017. Pharmacological targeting of adenosine receptor signaling. Mol Aspects Med 55:4–8. doi:10.1016/j.mam.2016.12.002 28088486

